# Supplementary material for: Evaluating phase synchronization methods in fMRI: A comparison study and new approaches
Source: Neuroimage. Author manuscript; Available in PMC 2021 Mar 31. (PMC8011682; doi:10.1016/j.neuroimage.2020.117704)
Supplement: 1 [file NIHMS1677103-supplement-1.pdf]

S1. Supplementary Materials

S1.1. Empirical Mode Decomposition

An important consideration when using measures of PS is to appropriately narrow-bandpass filter the data before applying the Hilbert transform. An alternative approach is to instead use empirical mode decomposition (EMD) (Huang, 2014). Here one decomposes the time series into a sum of oscillatory modes, referred to as intrinsic mode functions (IMF), that correspond to different frequency contents in the time series. EMD provides a data-driven signal decomposition that does not require an *a priori* defined basis system. The first IMF consists of the largest frequency oscillation present in the signal, and each subsequent IMF consists of increasingly lower frequency oscillations than those previously extracted.

The approach has recently found widespread usage in neuroimaging. For example, in an application to EEG data, Mutlu and Aviyente (2011) proposed using EMD to obtain a more robust representation of the time-varying phase synchronization across frequencies. Similarly, in applications to fMRI data Zhou et al. (2020) used EMD to predict and perform classification of sleep quality based on phase synchronization measured during resting state fMRI, while Goldhacker et al. (2018) used EMD followed by filter-bank to study frequency-resolved dynamic functional connectivity.

As we are primarily interested in bivariate measures of PS, we focus our attention on bivariate EMD (BEMD) (Rilling et al., 2007). Here EMD is performed jointly on the bivariate signal to ensure that the frequencies of the IMFs are matched to both signals. For simplicity, the bivariate signal is treated as a complex valued signal. In general, given a set of directions $\varphi_k = k\pi$ for $k = 1, 2$, the BEMD is performed as outlined in Algorithm 1.

We repeated Simulations 1-3 described in Section 2.3 using the BEMD-based phase synchronization of the generated signals. Results are shown in Figures S1 - S3 and described in the Sections 3.1 - 3.3.

Algorithm 1 Bivariate Empirical Mode Decomposition

1: **procedure** BEMD($x(t)$, STOPPAGE CRITERIA, NUMBER OF IMFS) ▷ $x(t)$ is the input signal
2: $\tilde{x}(t) \leftarrow x(t)$
3: **for** $1 \leq k \leq N$ **do**
4: Project the complex-valued signal $x(t)$ on direction φ_k:
5: $p_{\varphi_k}(t) = \mathbb{R}e\Big(e^{-i\varphi_k} x(t)\Big)$
6: Extract the maxima of $p_{\varphi_k}(t) : \{t_j^k, p_j^k\}$
7: Interpolate the set $\{(t_j^k, p_j^k)\}$ to obtain the "tangent" in direction
8: $\varphi_k : e'_{\varphi_k}(t)$
9: **end for**
10: Compute the mean of all tangents: $m(t) = \frac{2}{N} \sum_k e'_{\varphi_k}(t)$
11: $d(t) \leftarrow \tilde{x}(t) - m(t)$
12: $\tilde{x}(t) \leftarrow d(t)$ go to line 3; **repeat** until $d(t)$ becomes an IMF ▷ Stoppage Criteria
13: **repeat** lines 2:7 until desired number of IMFs obtained

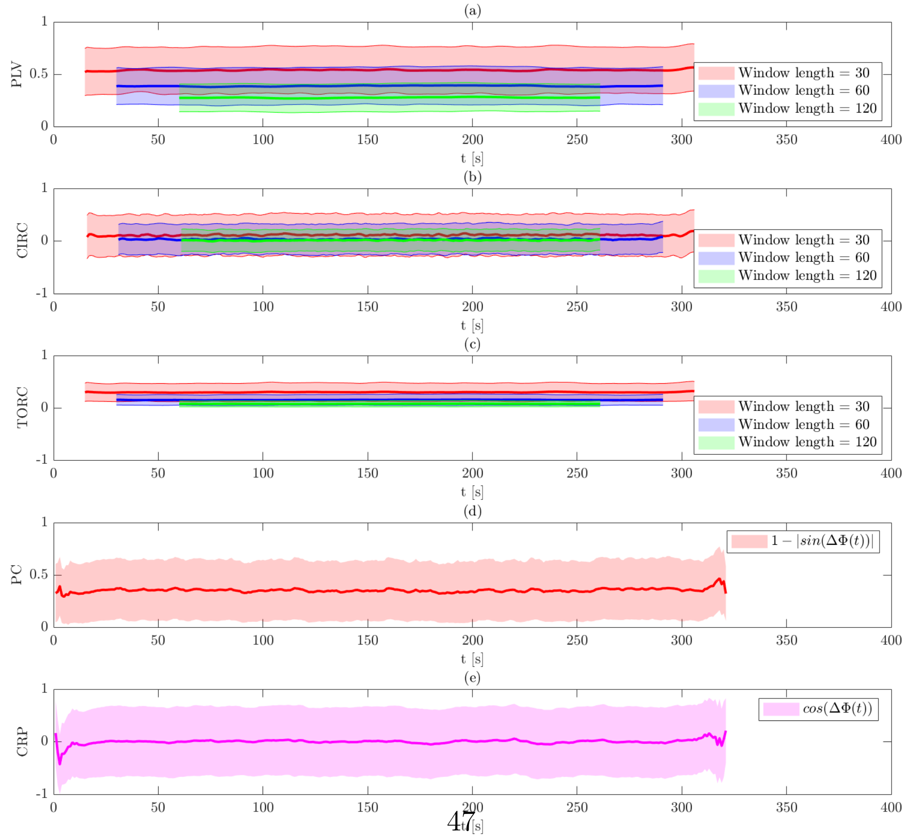

Figure S1: Results of Simulation 1 using BEMD-based PS. The bold line indicates the average estimated value, while the shaded area represents the 95% confidence interval. Results are shown for: (a) PLV using a sliding window; (b) circular-circular correlation using a sliding window; (c) toroidal correlation using a sliding window; (d) phase coherence; and (e) CRP. The sliding window techniques are evaluated at three different window lengths.

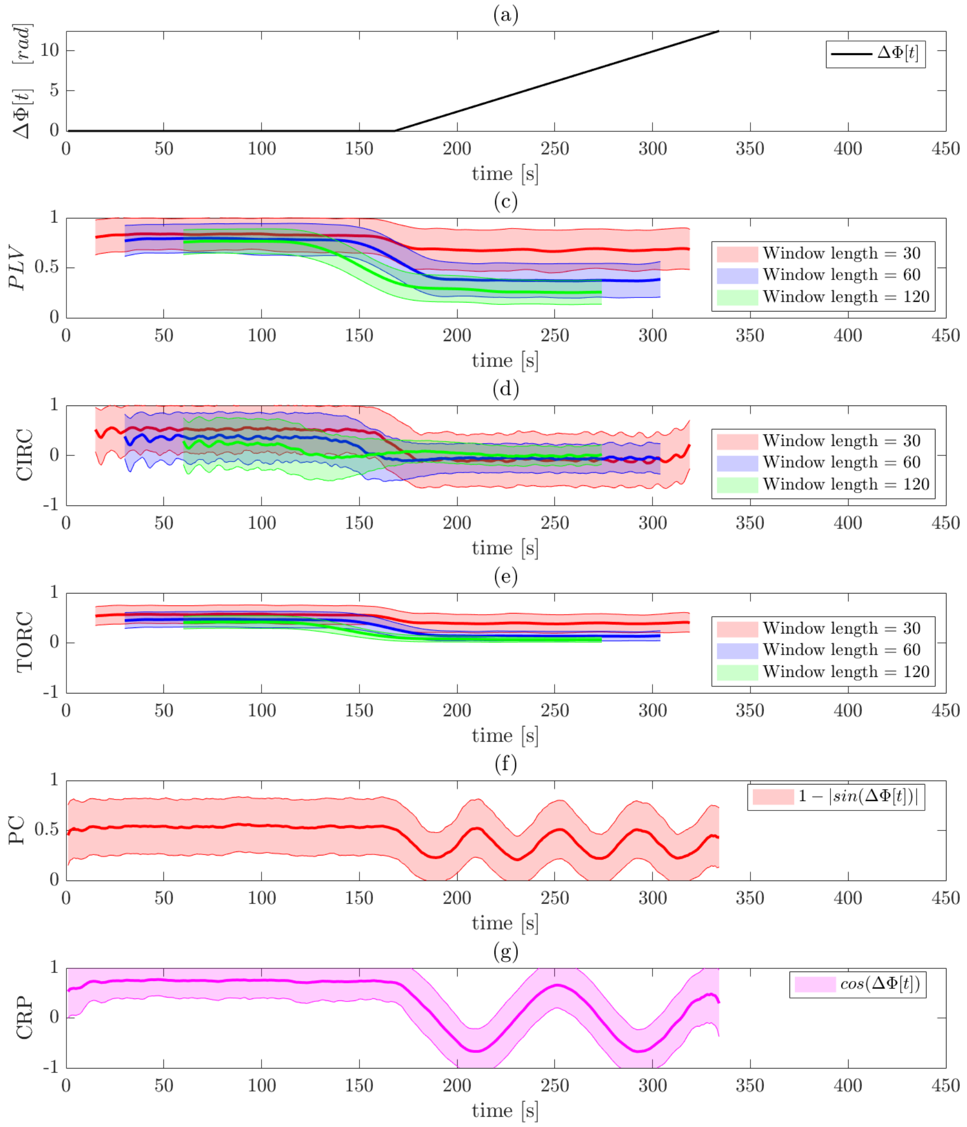

Figure S2: Results of Simulation 2 using BEMD-based PS. The bold line indicates the average estimated value, while the shaded area represents the 95% confidence interval. (a) The ground truth phase shift between the two signals as a function of time. Results are shown for: (b) PLV using a sliding window; (c) circular-circular correlation using a sliding window; (d) toroidal correlation using a sliding window; (e) phase coherence; and (f) CRP. The sliding window techniques are evaluated at three different window lengths.

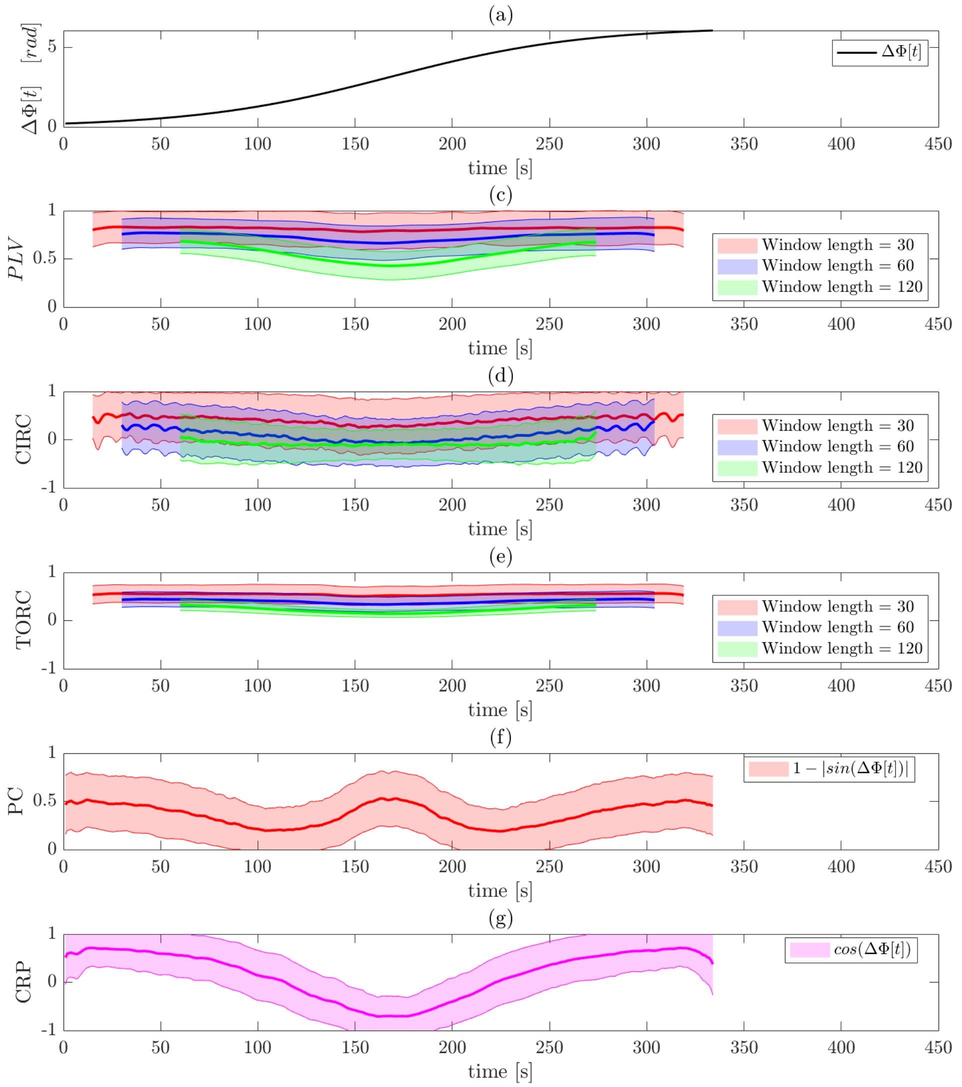

Figure S3: Results of Simulation 3 using BEMD-based PS. The bold line indicates the average estimated value, while the shaded area represents the 95% confidence interval. (a) The ground truth phase shift between the two signals as a function of time. Results are shown for: (b) PLV using a sliding window; (c) circular-circular correlation using a sliding window; (d) toroidal correlation using a sliding window; (e) phase coherence; and (f) CRP. The sliding window techniques are evaluated at three different window lengths.

S1.2. Extended simulation results

In this section, we present quantitative values for the bias and variance of the various PS measures estimated from Simulations 1 - 3. These values are provided as a supplement to Figures 3 - 8. To compute the bias, we calculated 'ground truth' values for the various methods based on the ground truth phase difference $\Delta\Phi_g(t)$ computed at each time point. For circular-circular correlation, toroidal correlation, and CRP we used the value $\cos(\Delta\Phi_g(t))$ as the ground truth. For PLV and PC, we used $1 - |sin(\Delta\Phi_g(t))|$ as the ground truth. For Simulation 1 we do not have measures of the bias for PLV and PC, as the appropriate null value is somewhat ambiguous. The values reported in Tables S1 - S3 are averages over time and repetitions of the simulation for each method. In the case of the bias, it is the average of the absolute value of the bias at each time point. To compute variances we estimated the variation in the repetitions around its mean value, and averaged across time.

No band-pass filtering						
**PS Measure**	**Bias**			**Variance**		
	**Sim 1**	**Sim 2**	**Sim 3**	**Sim 1**	**Sim 2**	**Sim 3**
PLV, w = 30	-	04550	0.2283	0.0115	0.0185	0.0186
PLV, w = 60	-	0.4801	0.2116	0.0060	0.0095	0.0106
PLV, w= 120	-	0.5015	0.2021	0.0029	0.0049	0.0056
PC	-	0.4361	0.2325	0.0945	0.0953	0.0945
CIRC, w= 30	0.0138	0.7837	0.6313	0.0346	0.0795	0.0849
CIRC, w=60	0.0063	0.8168	0.6116	0.0172	0.0460	0.0608
CIRC, w = 120	0.0052	0.8068	0.5475	0.0084	0.0303	0.0344
TORC, w = 30	0.0249	0.7680	0.6236	0.0027	0.0061	0.0066
TORC, w =60	0.0128	0.7925	0.5997	0.0007	0.0018	0.0023
TORC, w = 120	0.0064	0.7828	0.5353	0.0002	0.0006	0.0006
CRP	0.0177	0.5651	0.4671	0.4998	0.4380	0.4502

Table S1: The average bias and variance for each measure evaluated for each of the three simulations in the case where no band-pass filtering was performed.

Note we have separated the methods based on their range, as it is difficult to directly compare the bias and variance values across metrics that take different values and seek to measure different quantities. Generally, the bias

Band-pass filtering						
**PS Measure**	**Bias**			**Variance**		
	**Sim 1**	**Sim 2**	**Sim 3**	**Sim 1**	**Sim 2**	**Sim 3**
PLV, w = 30	-	0.2651	0.5408	0.0511	0.0208	0.0132
PLV, w = 60	-	0.2065	0.5149	0.0310	0.0162	0.0129
PLV, w= 120	-	0.2540	0.4380	0.0173	0.0081	0.0129
PC	-	0.2732	0.1118	0.0950	0.0723	0.0738
CIRC, w= 30	0.0843	0.4612	0.5187	0.1546	0.1472	0.0517
CIRC, w=60	0.0261	0.4529	0.3371	0.0809	0.0677	0.1082
CIRC, w = 120	0.0076	0.5895	0.3113	0.0414	0.0727	0.1913
TORC, w = 30	0.2563	0.5437	0.5858	0.0260	0.0328	0.0313
TORC, w =60	0.1295	0.5613	0.5581	0.0088	0.0132	0.0208
TORC, w = 120	0.0652	0.5975	0.5123	0.0026	0.0053	0.0084
CRP	0.0177	0.1487	0.1181	0.5060	0.1127	0.1503

Table S2: The average bias and variance for each measure evaluated for each of the three simulations in the case where band-pass filtering was performed.

EMD						
**PS Measure**	**Bias**			**Variance**		
	**Sim 1**	**Sim 2**	**Sim 3**	**Sim 1**	**Sim 2**	**Sim 3**
PLV, w = 30	-	0.2840	0.4758	0.0573	0.0386	0.0329
PLV, w = 60	-	0.2509	0.4425	0.0367	0.0293	0.0289
PLV, w= 120	-	0.2934	0.3949	0.0205	0.0178	0.0209
PC	-	0.3242	0.1444	0.0949	0.0860	0.0840
CIRC, w= 30	0.1273	0.5491	0.5299	0.1818	0.2853	0.3111
CIRC, w=60	0.0486	0.6800	0.4975	0.0970	0.1832	0.2927
CIRC, w = 120	0.0175	0.7509	0.5127	0.0504	0.1408	0.1630
TORC, w = 30	0.2959	0.5761	0.5799	0.0349	0.0378	0.0397
TORC, w =60	0.1510	0.6100	0.5591	0.0125	0.0189	0.0266
TORC, w = 120	0.0765	0.6397	0.5158	0.0036	0.0087	0.0100
CRP	0.0128	0.2553	0.2054	0.4956	0.2258	0.2391

Table S3: The average bias and variance for each measure evaluated for each of the three simulations in the case where EMD filtering was performed.

and the variance is lower for methods that take values in the range $[0, 1]$, compared with those that take values in the range $[-1, 1]$.

Studying the Tables, a few things standout. First, for each WPS method as the window length increases, the variance decreases. This is reasonable as additional data is used to calculate the metric of interest. Of the three WPS methods, TORC shows the lowest variance followed by PLV and CIRC. Again, note that PLV is measured on a different range than the other two measures which can complicate direct comparisons. Second, CRP reports consistently higher variance than the other methods (perhaps with the exception of CIRC). However, the bias is lower and it is clear from the figures that CRP is able to handle PS transitions better than any of the other methods. Hence, there is a bias-variance trade-off taking place which should be considered when choosing an appropriate measure. Finally, we found that when using the EMD-based phase synchronization approach, the results are consistent with those obtained using band-pass filtering. However, we found that the variance of the estimates obtained using the EMD-based phase synchronization framework is higher for the same level of noise.

S1.3. Re-analysis of Kirby data using different band-pass filters

We re-analyzed the Kirby data using a wider cutoff frequency band [0.01, 0.1] Hz as well as a number of different frequency bands [0.01, 0.05] Hz, [0.05, 0.1] Hz, and [0.1, 0.15] Hz respectively.

Figure S4 illustrates that using a wider frequency band ([0.01, 0.1] Hz) provides similar results to those shown in Figure 9, where a more narrow band ([0.03, 0.7] Hz) was used. While, the values in the estimated brain states are slightly lower, the overall structure is maintained. This is consistent with the results observed in the simulations performed without band-pass filtering. This behavior is particularly apparent for the WPS measures.

Figures S5 - S7 compare the results obtained using three different frequency bands: [0.01, 0.05] Hz, [0.05, 0.1] Hz, and [0.1, 0.15] Hz. In general, all bands appear to provide similar brain state estimates. Focusing on the results obtained for the band [0.05, 0.1] Hz, CRP yielded slightly lower estimates compared to those obtained using [0.01, 0.05] Hz and [0.03, 0.07] Hz. Using the band [0.1, 0.15] Hz yielded even lower estimates. Here the brain states estimated using CRP, took values closer to those obtained with PW-CSW.

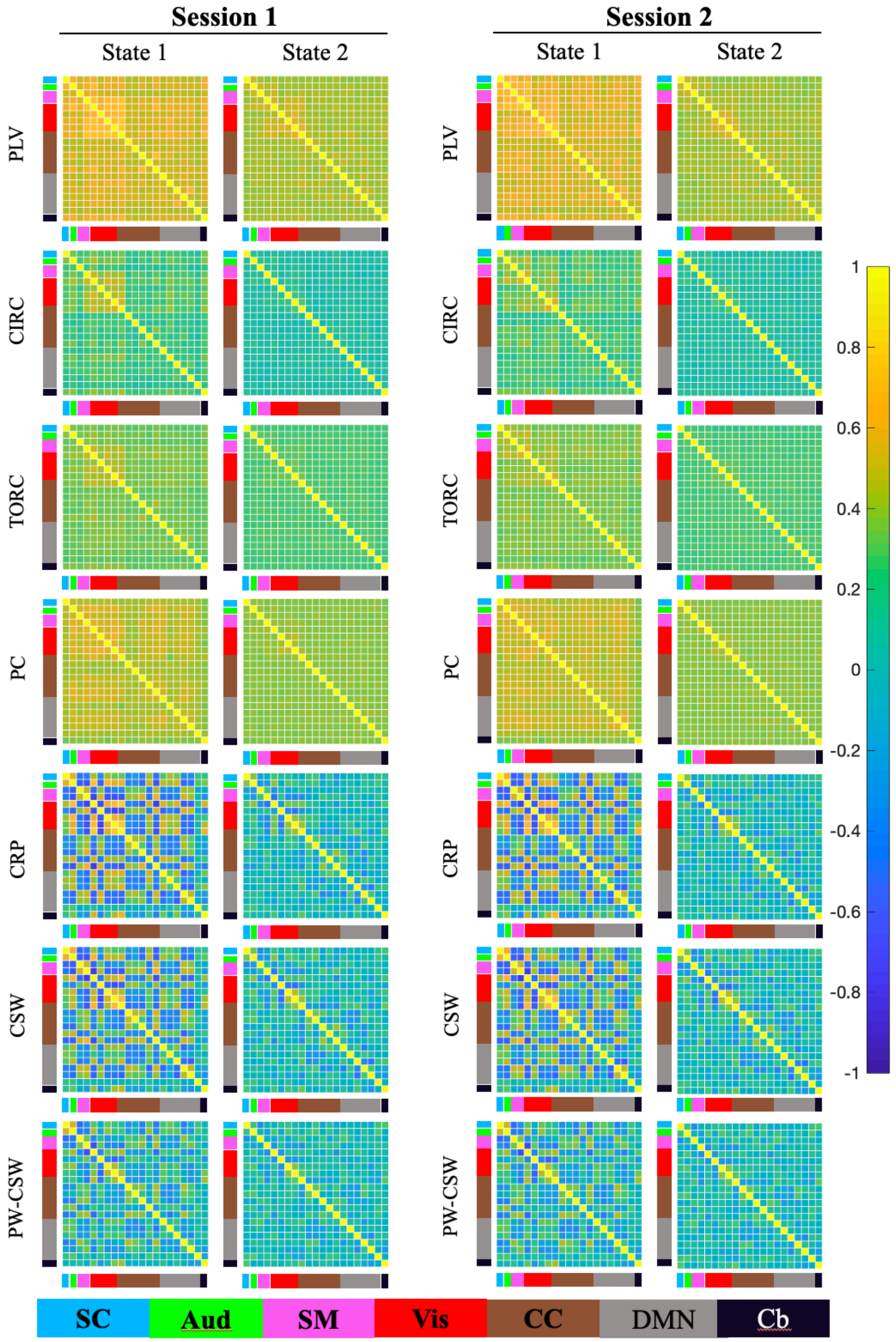

Figure S4: Re-analysis of the Kirby data using a wider cutoff frequency band $[0.01, 0.1]\ Hz$.

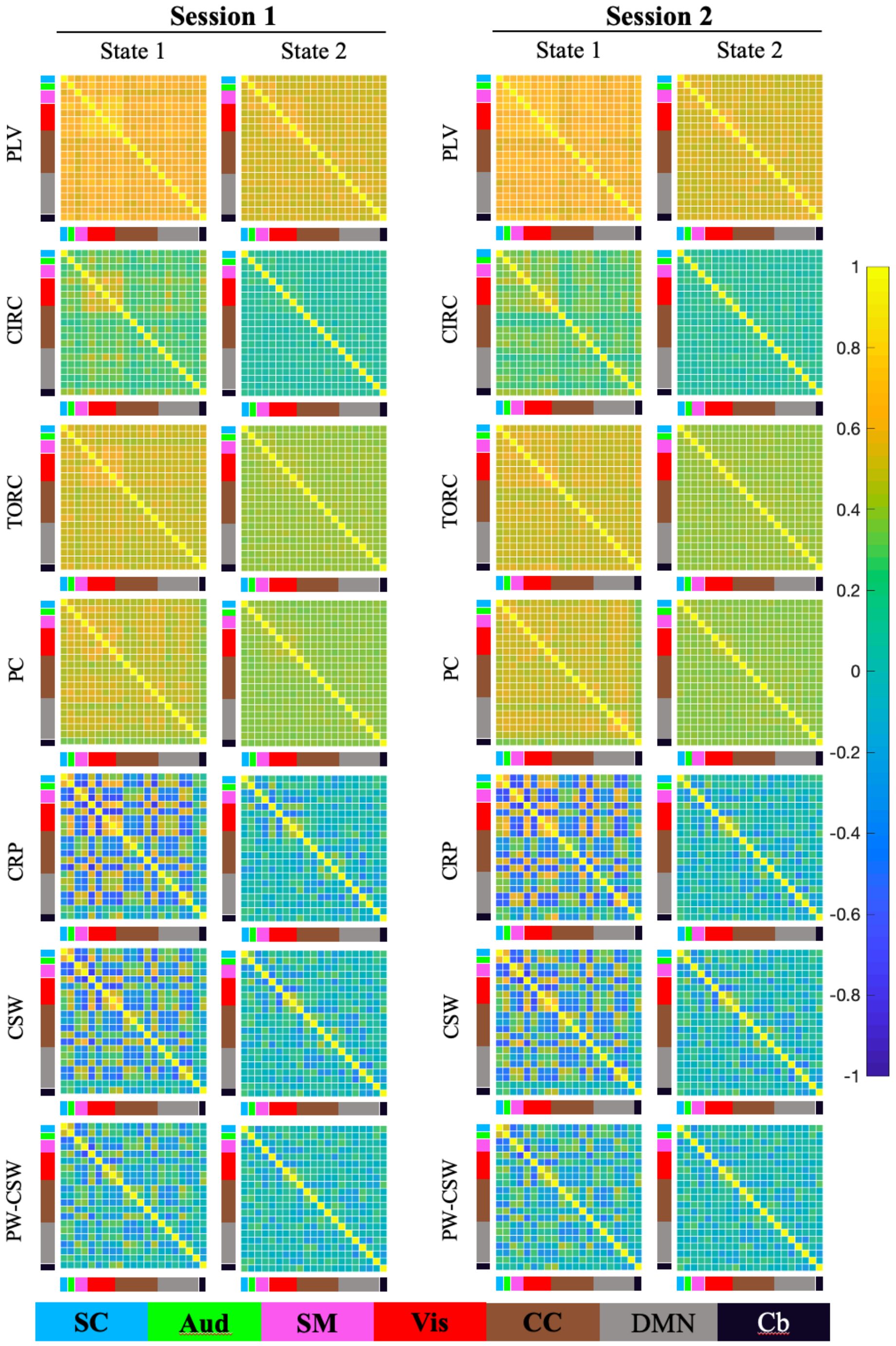

Figure S5: Analysis of the PS measures on Kirby21 Data using the cutoff frequency band $[0.01, 0.05]\,Hz$

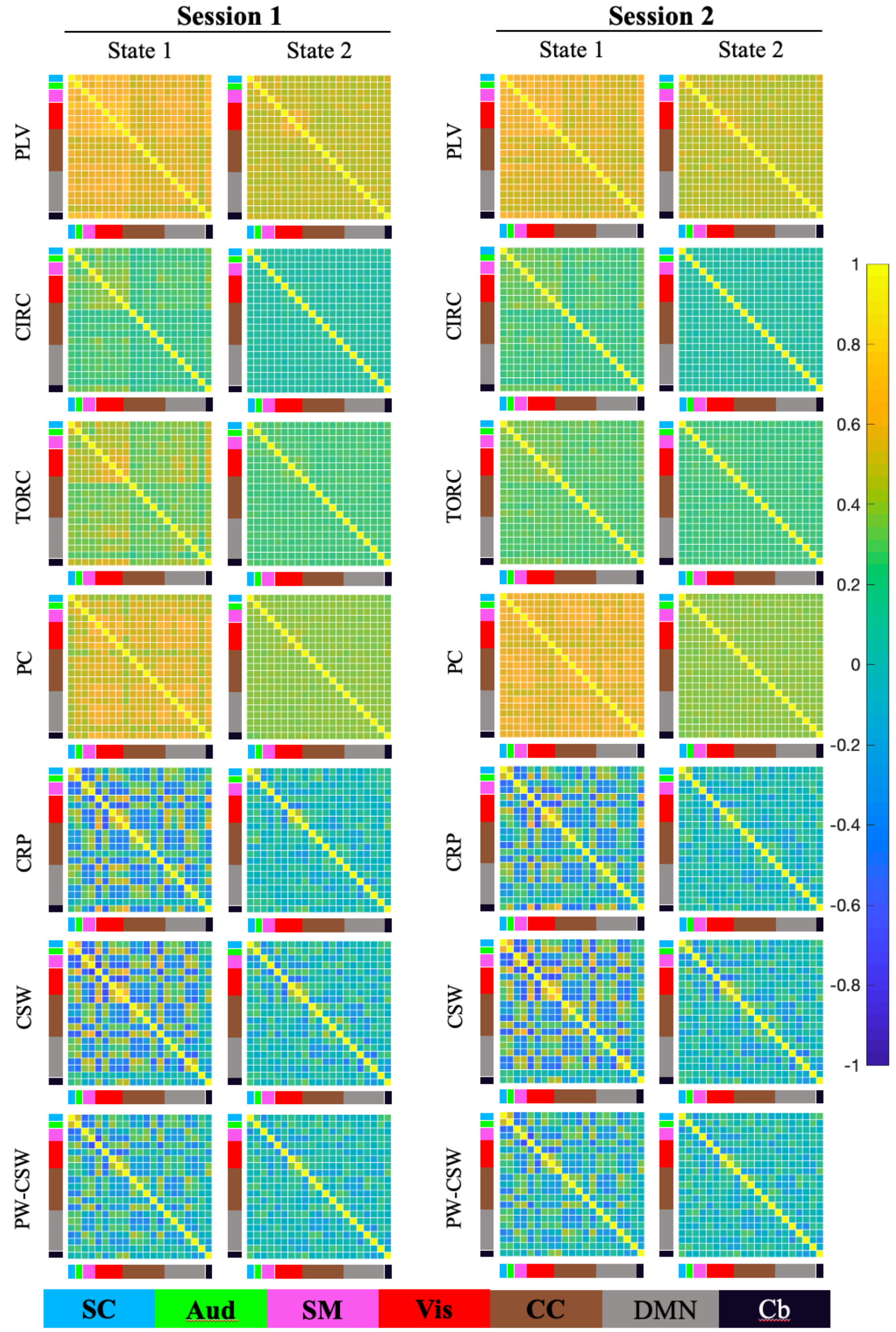

Figure S6: Analysis of the PS measures on Kirby21 Data using a wider cutoff frequency band $[0.05, 0.1]\,Hz$

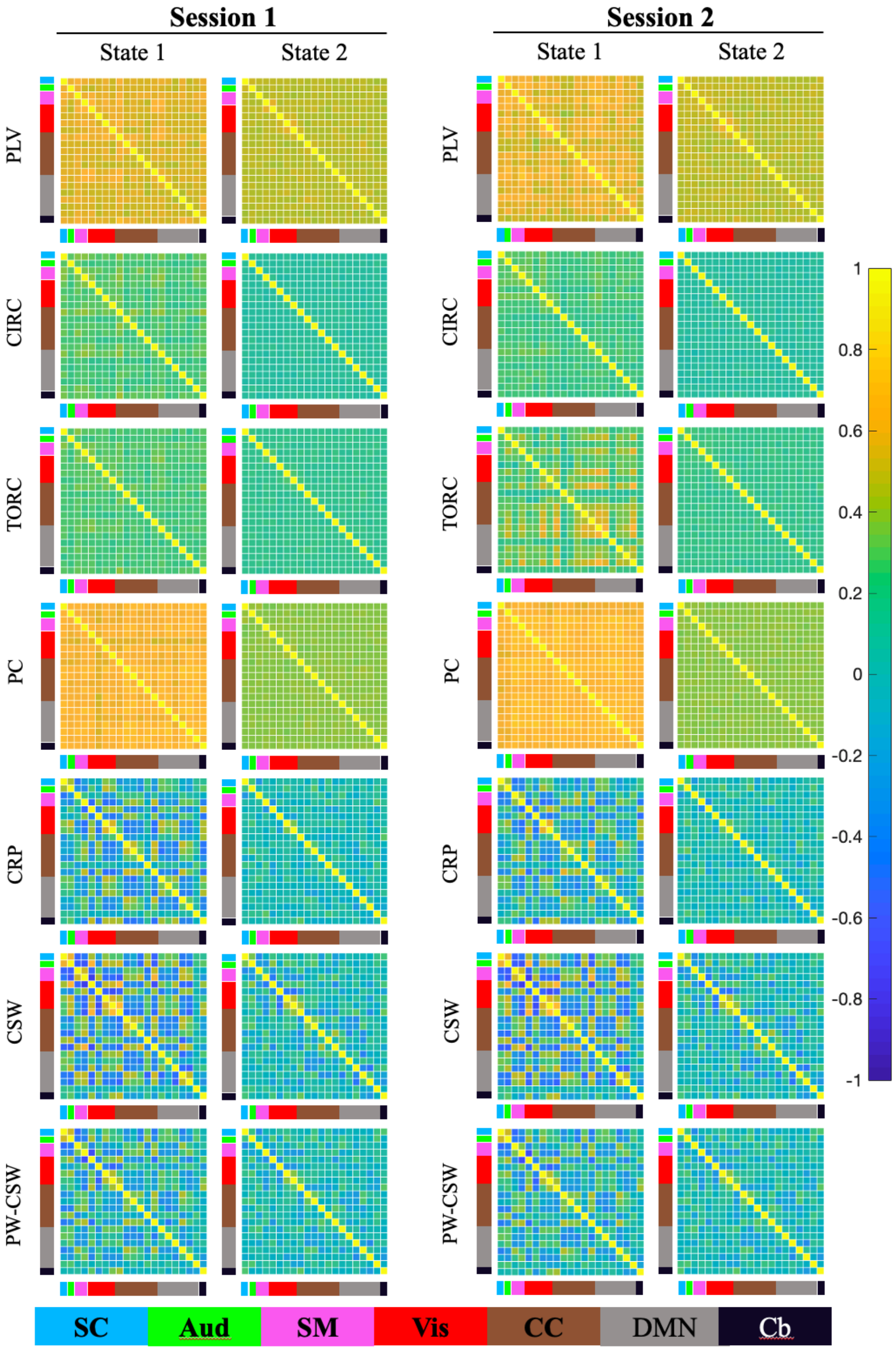

Figure S7: Analysis of the PS measures on Kirby21 Data using a wider cutoff frequency band $[0.1, 0.15]\, Hz$

S1.4. Application to HCP data

We repeated our analysis using rs-fMRI data from the 900-subject public data release from the Human Connectome Project (HCP) (Van Essen et al., 2013). For each subject, four 15 minute fMRI scans with a temporal resolution of 0.73 seconds and a spatial resolution of 2-mm isotropic were available. We used the preprocessed and artifact-removed rs-fMRI data provided through the HCP900-PTN data release. This data has been extensively described in multiple other publications, so we only briefly discuss it below.

The preprocessing pipeline followed the procedure outlined in Smith et al. (Smith et al., 2013). Spatial preprocessing was applied using the procedure described by Glasser et al. (2013). ICA, followed by FMRIBs ICA-based X-noisefier (FIX) from the FMRIB Software Library (FSL) (Griffanti et al., 2014), was used for structured artifact removal, removing more than 99 percent of the artifactual ICA components in the dataset. Group spatial ICA was then used to obtain a parcellation of 50 components that cover both the cortical surfaces and the subcortical areas. Global signal regression was not employed. The parcellation was used to project the fMRI data into 50 time series.

Following the same analysis pipeline as described in Section 2.4.3 we analyzed a single run consisting of 1200 time points for 50 subjects. We computed PS measures and used k-means clustering to estimate recurring brain states across subjects. Using the Davies-Bouldin Index we chose the number of centroids to represent four distinct brain states. Results are shown in Figure S8 below and described in the Results section.

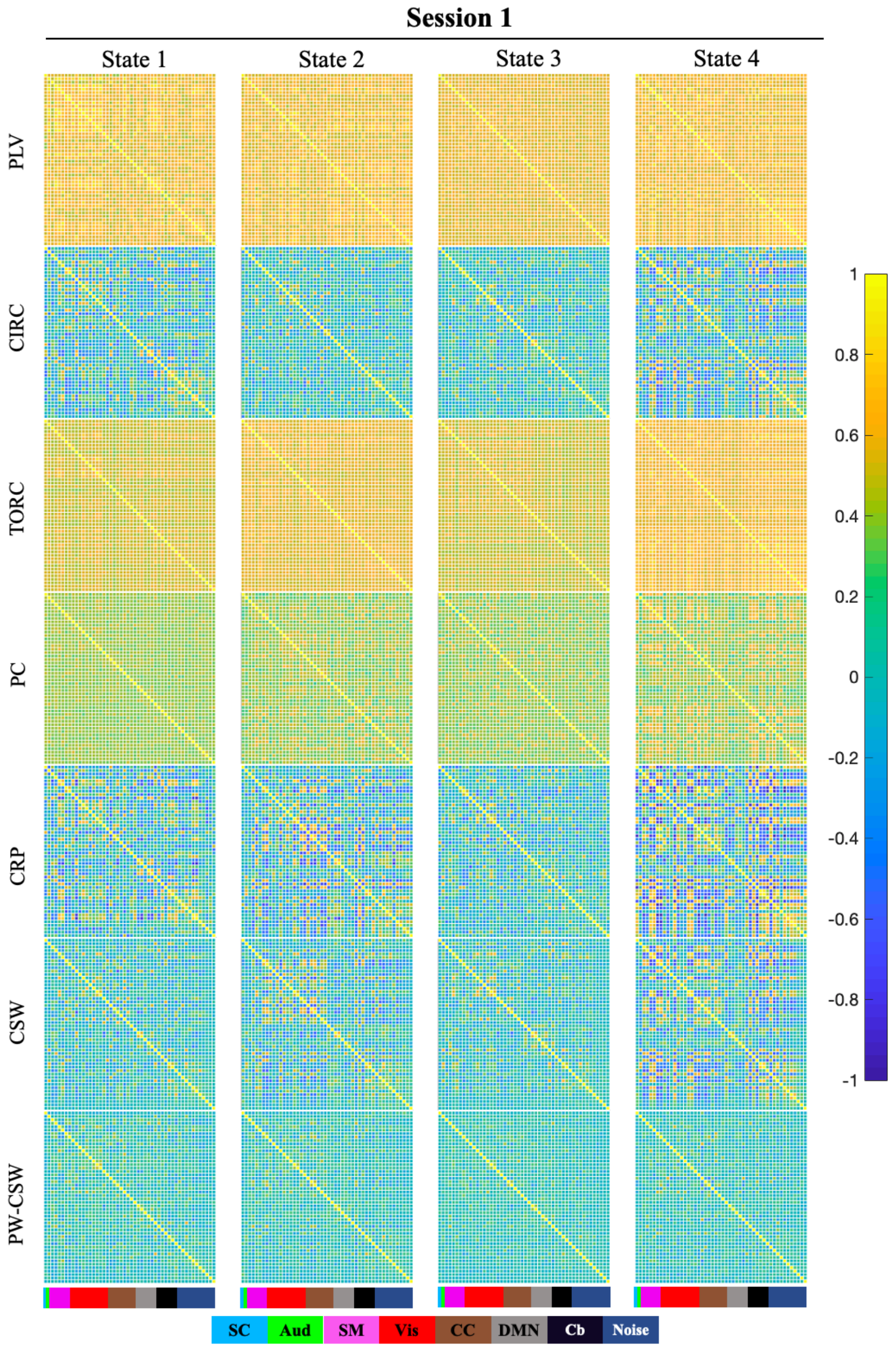

Figure S8: Comparison of the various phase syncrhonization measures on 50 subjects from HCP data
